# Why phylogenetic signal of traits is important in ecosystems: uniformity of a plant trait increases soil fauna, but only in a phylogenetically uniform vegetation

**DOI:** 10.1007/s00442-023-05384-z

**Published:** 2023-05-18

**Authors:** F. Molleman, N. Rossignol, J. F. Ponge, G. Peres, D. Cluzeau, N. Ruiz-Camacho, J. Cortet, C. Pernin, C. Villenave, A. Prinzing

**Affiliations:** 1 Department of Systematic Zoology, Faculty of Biology, Institute of Environmental Biology, Adam Mickiewicz University Poznań, Ul. Uniwersytetu Poznańskiego 6, 61-614 Poznań, Poland; 2 Université de Rennes 1/Centre National de la Recherche Scientifique, Research Unit ‘Ecobio-Ecosystèmes, Biodiversité, Evolution’, Campus Beaulieu, Bâtiment 14 A, 35042 Rennes, France; 3 Muséum National d’Histoire Naturelle, CNRS UMR 7179, 4 avenue du Petit Château, 91800 Brunoy, France; 4 UMR SAS INRAE Institut Agro Rennes-Angers, 65 Rue de St-Brieuc, 35042 Rennes, France; 5 Université de Rennes 1/Centre National de la Recherche Scientifique, Research Unit ‘Ecobio-Ecosystemes, Biodiversite, Evolution’, Station Biologique, 35380 Paimpont, France; 6 Agence Nationale de la Recherche, 50, avenue Daumesnil, 75012 Paris, France; 7 Centre d’Ecologie Fonctionnelle et Evolutive, Université Paul-Valéry Montpellier 3, Université de Montpellier, EPHE, IRD, Route de Mende, 34199 Montpellier, France; 8 Université de Lille, Institut Mines-Télécom, Université Artois, Junia, ULR 4515–LGCgE, Laboratoire de Génie Civil et geo-Environnement, 59000 Lille, France; 9 ELISOL environnement, ZA des Tourels, 10 avenue du midi, 30111 Congénies, France

**Keywords:** Abundance, Diversity, Decomposer, Functional traits, Grassland, Phylogenetic diversity, Resource concentration, Resource dilution

## Abstract

Phylogenetically closely related plant species often share similar trait states (phylogenetic signal), but local assembly may favor dissimilar relatives and thereby decouple the diversity of a trait from the diversity of phylogenetic lineages. Associated fauna might either benefit from plant trait diversity, because it provides them complementary resources, or suffer from it due to dilution of preferred resources. We hence hypothesize that decoupling of trait and phylogenetic diversity weakens the relationship between the plant-trait diversity and the abundance and diversity of associated fauna. Studying permanent meadows, we tested for combined effects of plant phylogenetic diversity and diversity of two functional traits (specific leaf area, leaf dry matter content) on major groups of soil fauna (earthworms, mites, springtails, nematodes). We found that only in phylogenetically uniform plant communities, was uniformity in the functional traits associated with (i) high abundance in springtails, and (ii) high abundance of the sub-group that feeds more directly on plant material (in springtails and mites) or those that are more prone to disturbance (in nematodes), and (iii) high diversity in all three groups tested (springtails, earthworms, nematodes). Our results suggest that soil fauna profits from the resource concentration in local plant communities that are uniform in both functional traits and phylogenetic lineages. Soil fauna would hence benefit from co-occurrence of closely related plants that have conserved the same trait values, rather than of distantly related plants that have converged in traits. This might result in faster decomposition and a positive feedback between trait conservatism and ecosystem functioning.

**Supplementary Information:**

The online version contains supplementary material available at 10.1007/s00442-023-05384-z.

## Introduction

Phylogenetically closely related plant species often share similar trait states (Peterson 2011), even locally. However, in disturbed habitat types like meadows in temperate regions, local phylogenetic signal of traits may be weak (Prinzing et al. 2021). This pattern of low phylogenetic signal suggests that local assembly favors distant relatives that converged in trait states or close relatives that diverged. Such convergent or divergent trait states exist because during diversification, trait evolution was sometimes labile (Ackerly 2004; Grime 2006). For simplicity, we hence below refer to the pattern of low local phylogenetic signal as “trait lability”, acknowledging that this pattern results from local assembly of species and traits that have evolved elsewhere. As a result of such local trait lability, the local diversity of a given functional trait may be decoupled from the local phylogenetic diversity: local communities will sometimes be diverse in states of a given trait but uniform in phylogenetic lineages, or uniform in trait states but diverse in lineages (Losos 2008). Local diversity of a plant trait may, in turn, affect associated fauna (Beugnon et al. 2019), but we do not know whether this effect depends on whether the trait diversity is coupled with phylogenetic diversity. We will below develop how local diversity of a trait may affect associated fauna, and then how this effect may depend on coupling of this trait to phylogenetic diversity.

Local plant communities that have a large diversity of trait states may provide a large diversity of resources to associated fauna, thereby increasing the abundance and diversity of animals due to increased complementarity among resources (Eisenhauer 2012). For instance, generalist folivorous Orthoptera can balance their diet by feeding on multiple plant species and are worse than specialists at coping with feeding on a single plant species that provides a non-balanced diet (Raubenheimer and Simpson 2003). On the other hand, if the diversity of resources is large, none of them is abundant, so that the preferred resources for any given animal species are diluted, potentially reducing their abundance or even preventing their subsistence (Root 1973). The diversity of resources for animals within a local plant community has often been inferred from the diversity in particular key functional traits, each being supposed to be locally related to many other traits through evolutionary conserved “economic spectra” (e.g. Flores et al. 2014; Jardine et al. 2020; Li et al. 2017).

**Fig. 1:**
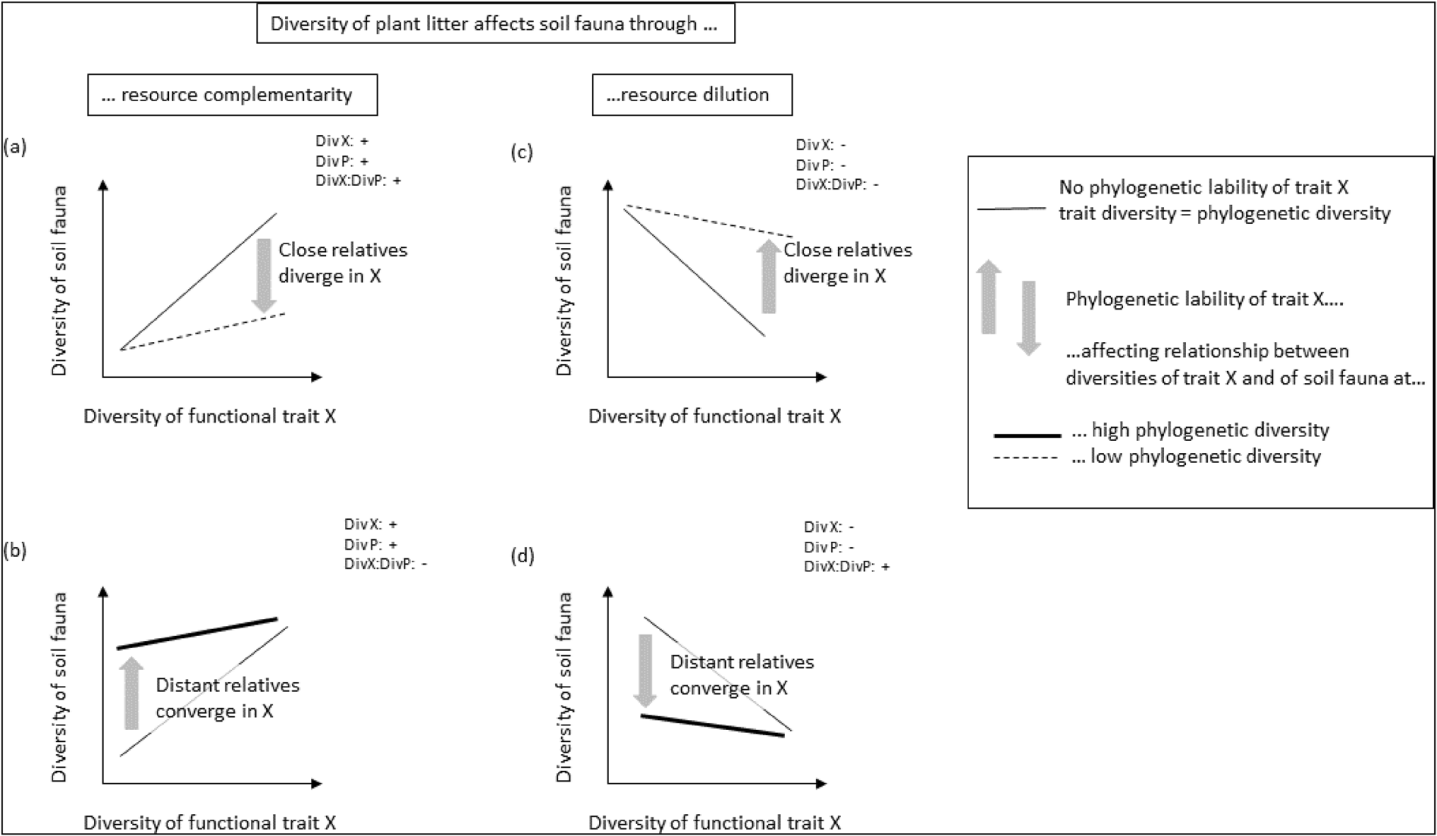
How the phylogenetic diversity of plants and the diversity of a functional plant trait may interact to determine abundance of associated animals. Abundance of animals is favored by the diversity of plant resources through complementarity among litters (**a**, **b**), or reduced through dilution of preferred resources for any individual animal species (**c**, **d**). High or low diversity for a given functional trait X (X-axis) corresponds to high or low phylogenetic diversity if X locally shows phylogenetic signal (thin straight line). However, if among the locally represented species the trait X was evolutionary labile (gray arrows), we may expect locally high diversity of X also through past divergence of close relatives (dotted lines in **a** and **c**), or locally low diversity of X also through past convergence among distant relatives (thick straight lines in **b**, **d**). High diversity of X among otherwise similar, closely related plant species may imply less resource complementarity or dilution than high diversity of X among distantly related species (inversely for low diversity of X through convergence among distantly related species). Div X, Div P, and DivX:DivP represent expected effects of diversity of functional litter trait X, phylogenetic diversity, and their interaction on faunal diversity, respectively. The interaction term hence describes how the evolutionary lability of an individual plant trait changes the relationship between the diversity of that trait and the diversity of associated animals. Similar relationships may be posited for animal abundance

However, when in a local plant community, a given trait is evolutionary labile, the diversity of that trait does not coincide with high phylogenetic diversity or the diversity of other conserved traits (Tucker et al. 2018). First, the local plant community may be composed of closely related species that diverged in this particular trait but remained similar in many other traits. We hypothesize that due to this similarity, the abundance and diversity of fauna may neither increase due to complementarity, nor decrease due to resource dilution (Fig. 1a, c). As an extreme example, a large diversity of plant sizes represented by phylogenetically diverse Fabaceae, Poaceae, Salicaceae, and Fagaceae on a continent may produce more complementary (or more diluted) resources than the same diversity of plant sizes represented by phylogenetically closely related Boraginaceae species on an oceanic island (Nürk et al. 2019). Second, the local plant community may be composed of distantly related species that converged in this functional trait but remained different in many other functional traits (Fig. 1b, d). We hypothesize that in that case, resources for animals might be complementary (or diluted) despite low diversity in this particular trait, because other traits are different. Overall, we predict that the evolutionary lability of a plant trait alters the relationship between the diversity of that trait and the diversity and abundance of soil fauna (arrows in Fig. 1). This altered relationship results in a statistical interaction term between phylogenetic and functional diversity on soil fauna (different types of lines in Fig. 1).

The effect of phylogenetic position and functional traits of plants on associated fauna has been studied mostly for phytophages feeding on living plants, and rarely for soil animals living in dead litter. For phytophages, it has been shown that evolutionary histories of plant lineages are intimately related to co-evolutionary histories of associated phytophagous arthropods: many species of phytophages feed only on a small number of closely related plant species, probably mediated in part by phylogenetic signals in leaf quality (Brändle and Brandl 2006). Consequently, the phylogenetic diversity of a local plant community has major effects on the diversity, abundance, and trophic structure of its phytophagous arthropod community (Jactel et al. 2005; Jactel and Brockerhoff 2007; Molleman et al. 2022; Schuldt et al. 2019; Vialatte et al. 2010; Yguel et al. 2011). Furthermore, it has been shown that the Specific Leaf Area (SLA) and Leaf Dry Matter Content (LDMC) of a leaf may determine which phytophages can use it (Bisigato et al. 2015; Castagneyrol et al. 2017; Descombes et al. 2017; Schädler et al. 2003; Schuldt et al. 2012), and higher diversity in such traits may increase the diversity of phytophagous arthropods (Marini et al. 2009).

In contrast, it has been much less studied how local plant traits or the phylogenetic clades to which plants belong affect soil fauna. Contrary to phytophagous arthropods, many soil organisms feed on dead plant material which has been previously conditioned by microbes, thus with much less active plant defences. Furthermore, leaf litter is typically mixed at small spatial scales so that individual soil animals are more likely to have mixed diets than for example the most often studied folivores, Lepidopteran larvae. Finally, soil animals are usually hidden from view so that there is no selection for visual crypsis which promotes host-plant specialization in folivores (Lichter-Marck et al. 2015). Nevertheless, plant functional traits have been shown to affect leaf-litter traits and hence soil fauna (Eisenhauer and Powell 2017). In particular, structure-related traits such as captured in SLA and LDMC are important to decomposition and soil fauna, as they play an important role in determining the nutritional quality of leaf litter (Cornelissen 1996; Lin et al. 2019; Zukswert and Prescott 2017). Furthermore, some after-life traits of plant litter that affect soil fauna show phylogenetic signal (Cornelissen 2004; Grime et al. 1996; Pan et al. 2015a; Pan et al. 2015b). As a result, increased phylogenetic diversity of local plant communities should be accompanied by increased diversity in after-life traits of litter. Examples of specialization in soil biota on particular plant lineages include springtails becoming more abundant with the establishment of gymnosperms (Luque et al. 2011), soil-mediated interactions being strongest among closely related plant species (Anacker et al. 2014), fungus gnats being moderately specialized on fungus lineages (Põldmaa et al. 2016), and particular soil microbes associating with particular plant species (Leff et al. 2018).

Local plant communities that are diverse might produce leaf litter that is nutritionally complementary or diluted, thereby potentially favoring or disfavouring decomposer fauna, depending on their degree of specialization. For example, if plant species with divergent functional traits are nutritionally complementary, then a given generalist decomposer species in a diverse plant community may be able to balance its diet using the complementarity of material from different plant lineages. Such effects of complementarity among plant species on decomposer fauna may explain results of experiments in which diverse litter mixtures decomposed faster than predicted from decomposition rates of litter from single species (Bila et al. 2014; Gessner et al. 2010; Meier and Bowman 2008; Rabelo et al. 2022; Tardif and Shipley 2014; Vos et al. 2013). If, however, decomposer fauna is composed of specialist species that are good at coping with nutritional challenges of the litter of particular plant species, decomposer species might suffer from dilution of preferred resources under high phylogenetic or functional trait diversity (Barbe et al. 2018; Pan et al. 2015a; Plazas-Jiménez and Cianciaruso 2021). Overall, in local plant communities that are diverse, a species of generalist decomposer might do well, because it benefits from resource complementarity (Eisenhauer 2012), while a species of specialist decomposer might suffer from resource dilution (Root 1973). Therefore, both low and high phylogenetic or functional diversity of a local plant community might increase the abundance and diversity of soil fauna, depending on its degree of dietary specialization. In addition, high functional or phylogenetic diversity may support more diverse communities of specialists (Chesson 2000; Clavel et al. 2011; Jactel et al. 2021; Procheş et al. 2009). While local phylogenetic or functional diversity of plant communities has been shown to relate to decomposition rates (Barbe et al. 2017, 2018; Chamagne et al. 2016), its relationship to soil fauna has been very little studied. Milcu et al. (2013) found little effect of plant phylogenetic diversity on macroscopic decomposers in experimental plant communities, and positive effects on soil microbial biomass. Interacting effects of diversities of phylogenetic lineages and a given functional trait of plants on soil fauna (such as the hypotheses in Fig. 1) have to our knowledge never been studied. Overall, further studies are needed on effects of plant phylogenetic diversity and diversity of individual functional traits, and their interaction, on multiple classes of soil organisms in naturally assembled plant communities.

We tested the predictions in Fig. 1 on relationships between phylogenetic diversity and the diversity of a given key functional trait of local plant communities and soil fauna, considering meadows in Brittany, France. For each plant community, we first calculated the diversity of phylogenetic lineages and of two functional traits known to be major determinants of decomposition—SLA, and LDMC (Cornelissen 1996; Cornelissen 2004; Lin 2019). We tested for associations between plant phylogenetic and functional diversity and the abundance of earthworms, nematodes, springtails, and mites. Within each group, we differentiated sub-groups that are likely to be more exposed to plant diversity from those that are less exposed. Exposure may be due to spatial proximity (epigeic earthworms and hemiedaphic springtails being more exposed than endogeic earthworms and eudaphic springtails), trophic proximity (plant-feeding nematodes and mites vs. carnivorous nematodes and mites), or long life span and disturbance sensitivity (summarized by nematode community indices; Bouché 1972; Ferris et al. 2001; Gisin 1943; Walter and Proctor 2013). We also considered the diversity of three major soil fauna groups; earthworms, nematodes, and springtails (mites were not identified to species). We used the two functional traits in separate models with as dependent variable the various soil fauna parameters (Table 1). To test our hypotheses (Fig. 1), we were especially interested in the statistical interaction between plant phylogenetic diversity, and the diversity of functional traits.

**Table 1:** Relationships between soil fauna and phylogenetic and functional-trait diversity of plant communities (PhylDiv, FunTraitDiv) and their interactions

Functional trait	Soil fauna group	N sites	VegData	PhylDiv	meanFunTrait	FunTraitDiv	PhylDiv*FunTraitDiv	*R*^2^	*R*^2^ adj
(a) Abundance of major soil fauna groups									
SLA	Mites	12	0.54 (0.61)	0.13 (0.90)	0.30 (0.78)	0.72 (0.50)	0.22 (0.83)	0.21	− 0.44
	Springtails	15	0.10 (0.92)	− 0.75 (0.47)	− 1.01 (0.09)	− 0.02 (0.98)	2.06 (0.07)	0.53	0.27
	Earthworms	19	**2.75 (0.02)**	− 1.25 (0.23)	1.14 (0.27)	− 1.53 (0.15)	0.57 (0.58)	0.60	0.44
	Nematodes	19	− 1.58 (0.14)	0.84 (0.42)	0.85 (0.41)	1.60 (0.13)	0.33 (0.74)	0.35	0.10
LDMC	Mites	12	0.83 (0.44)	1.26 (0.25)	1.04 (0.34)	− 0.83 (0.44)	1.60 (0.16)	0.35	− 0.18
	Springtails	15	0.89 (0.40)	1.17 (0.27)	− 1.03 (0.33)	− 1.76 (0.11)	2.15 (0.06)	0.50	0.21
	Earthworms	19	− **2.37 (0.03)**	− 0.67 (0.52)	− 0.28 (0.78)	− 0.70 (0.49)	0.73 (0.48)	0.54	0.36
	Nematodes	19	− 1.37 (0.19)	0.05 (0.96)	0.84 (0.42)	1.35 (0.20)	0.94 (0.37)	0.34	0.09
(b) Abundance per earthworm group									
SLA	Epigeic	19	0.82 (0.43)	0.2 (0.85)	− 0.59 (0.56)	− 1.42 (0.18)	0.88 (0.40)	0.39	0.15
	Endogeic	18	**2.22 (0.04)**	− 1.91 (0.29)	0.79 (0.44)	− 0.49 (0.63)	0.39 (0.70)	0.41	0.18
LDMC	Epigeic	17	− 0.30 (0.77)	0.63 (0.54)	0.27 (0.79)	− **2.45 (0.03)**	1.33 (0.21)	0.45	0.23
	Endogeic	19	− 2.00 (0.07)	− 0.62 (0.55)	0.02 (0.98)	− 0.10 (0.92)	0.52 (0.61)	0.39	0.15
(c) Abundance per springtail group									
SLA	Hemiedaphic	14	0.72 (0.49)	0.69 (0.51)	1.01 (0.34)	− 0.71 (0.50)	**2.45 (0.04)**	0.53	0.24
	Eudaphic	14	0.47 (0.65)	− 0.87 (0.41)	− 0.28 (0.79)	0.21 (0.84)	0.23 (0.82)	0.17	− 0.35
LDMC	Hemiedaphic	14	2.02 (0.08)	2.21 (0.06)	− 1.36 (0.21)	− 2.01 (0.08)	**2.45 (0.04)**	0.56	0.29
	Eudaphic	14	0.18 (0.86)	− 1.08 (0.59)	0.06 (0.95)	0.52 (0.62)	− 0.58 (0.58)	0.20	− 0.30
(d) Abundance per mite group									
SLA	Actenida	13	0.77 (0.47)	1.38 (0.21)	− 0.92(0.39)	1.08 (0.32)	**2.45 (0.04)**	0.63	0.36
	Gamasida	15	− 1.51 (0.16)	− 0.65 (0.53)	1.04 (0.33)	0.33 (0.75)	0.62 (0.55)	0.34	− 0.02
LDMC	Actenida	13	1.34 (0.22)	2.07 (0.08)	0.30 (0.77)	− 0.64 (0.54)	2.05 (0.08)	0.48	0.10
	Gamasida	15	− 0.66 (0.52)	0.69 (0.51)	1.38 (0.20)	− 0.97 (0.36)	1.92 (0.09)	0.48	0.20
(e) Abundance of nematodes per feeding guild									
SLA	Phytopahages	19	**2.20 (0.05)**	− 0.90 (0.39)	0.80 (0.44)	1.87 (0.08)	− 0.13 (0.89)	0.39	0.16
	Carnivores	19	1.23 (0.24)	**2.53 (0.03)**	1.39 (0.19)	0.26 (0.80)	**2.33 (0.04)**	0.59	0.43
LDMC	Phytopahages	19	− 1.97 (0.07)	− 0.34 (0.74)	0.78 (0.45)	1.60 (0.13)	0.28 (0.78)	0.33	0.06
	Carnivores	19	0.90 (0.39)	1.59 (0.14)	0.96 (0.35)	1.53 (0.15)	0.95 (0.36)	0.54	0.36
(f) Nematode Structure Index accounting for abundances of ling lived vs short-lived species									
SLA		19	− 1.19 (0.25)	**2.56 (0.02)**	0.28 (0.78)	0.74(0.47)	**3.04 (0.01)**	0.53	0.35
LDMC		19	− 0.82 (0.43)	1.53 (0.15)	0.14 (0.89)	1.14 (0.28)	0.91 (0.38)	0.32	0.06
(g) Simpson diversity									
SLA	Earthworms	19	1.43 (0.18)	− 0.39 (0.71)	0.66 (0.52)	− 0.37 (0.72)	0.96 (0.35)	0.24	− 0.05
	Springtails	13	0.11 (0.92)	1.68 (0.14)	− 0.67 (0.52)	− 0.02 (0.99)	**2.64 (0.03)**	0.56	0.25
	Nematodes	18	**6.73 (< 0.01)**	**4.68 (< 0.01)**	0.36 (0.72)	− **3.21 (0.01)**	**3.65 (< 0.01)**	0.86	0.80
LDMC	Earthworms	19	− 1.00 (0.34)	0.81 (0.43)	0.07 (0.94)	− 0.72 (0.48)	1.90 (0.08)	0.35	0.10
	Springtails	13	0.81 (0.44)	**2.63 (0.03)**	− 0.75 (0.48)	− 0.25 (0.81)	**2.43 (0.05)**	0.52	0.18
	Nematodes	18	**3.84 (< 0.01)**	1.65 (0.13)	− 0.82 (0.43)	− 0.61 (0.55)	0.59 (0.56)	0.63	0.48

Soil fauna is the dependent variable and is characterized by Simpson diversity (a) and abundance (b) of major soil fauna groups, and abundances of sub-groups expected to be strongly or weakly exposed to plant diversity due to spatial position (c, d), trophic position (e, f) or life span (g)

Analyses separated for groups expected to be strongly or weakly exposed to plant diversity due to spatial (c, d) or trophic (e–f) position or life span (g)

The functional traits are specific leaf area (SLA) and leaf dry matter content (LDMC). Relationships between independent and dependent variables are quantified as *t* values (and *p* values in brackets). Significant (*p* < 0.05) relationships are in bold, and marginally significant (0.05 ≤ *p* < 0.1) are underlined. Phylogenetic diversity was calculated as Abu.mpd (abundance-weighted mean pairwise phylogenetic distance). Covariates are the source of the plant community data (VegData) and community-weighted mean of the respective functional trait (meanFunTrait), as defined in Methods. The number of sites varied between soil fauna groups due to missing data and the removal of outliers

## Methods

### Description of local plant communities

The plant community data were collected as part of the ‘Réseau de Mesures de la Qualité des Sols’ (RMQS), a campaign to systematically sample and analyze soils across France. When these plant community data were not available, we used those from RMQS-BioDiv. RMQS-BioDiv is a research program based at the University of Rennes 1 that provided data on soil fauna from the RMQS sites in the French Region of Brittany (Ponge et al. 2013). We selected all 19 sites with permanent meadows that were sampled.

RMQS plant community data were collected with a variant of the point-centred quarter method, originally developed for forest plots (Cotham and Curtis 1956) and also applied to grassland communities (Dix 1961). Classically, in each of the four cardinal directions, the distance of the first individual of each species to the central point is measured. In RMQS, instead of using the central point, the points at the corners of a 20 × 20 m sampling plot were taken as bases for distance measurements in all directions, extending to 3.5 m from each of the four points. The density of a given plant species was approximated by the inverse of the square of the average distance to the point (1/d^2^), reflecting that plant species encountered closer to the observation points tend to be more abundant. In case of an average distance of zero across the four corners, it was substituted by one cm. Subsequently, we multiplied this ‘average density’ by its frequency in the four corners, where the absence in a corner was regarded as a density of zero. We then scaled the resulting densities so that the total was one within sites, which is comparable to proportional abundance. For technical reasons, the plant community of six of the sites was not determined by RMQS, but was determined by RMQS-BioDiv using cover estimates. Both methods provide measures of plant cover per species, which in grasslands should correlate reasonably well with litter biomass produced (Röttgermann et al. 2000). We used RMQS data to characterize sites by the soil properties: humus index, waterlogging, soil depth, organic carbon, total nitrogen, C/N ratio, and water pH (Arrouays et al. 2002; Terrat 2017).

**Fig. 2:**
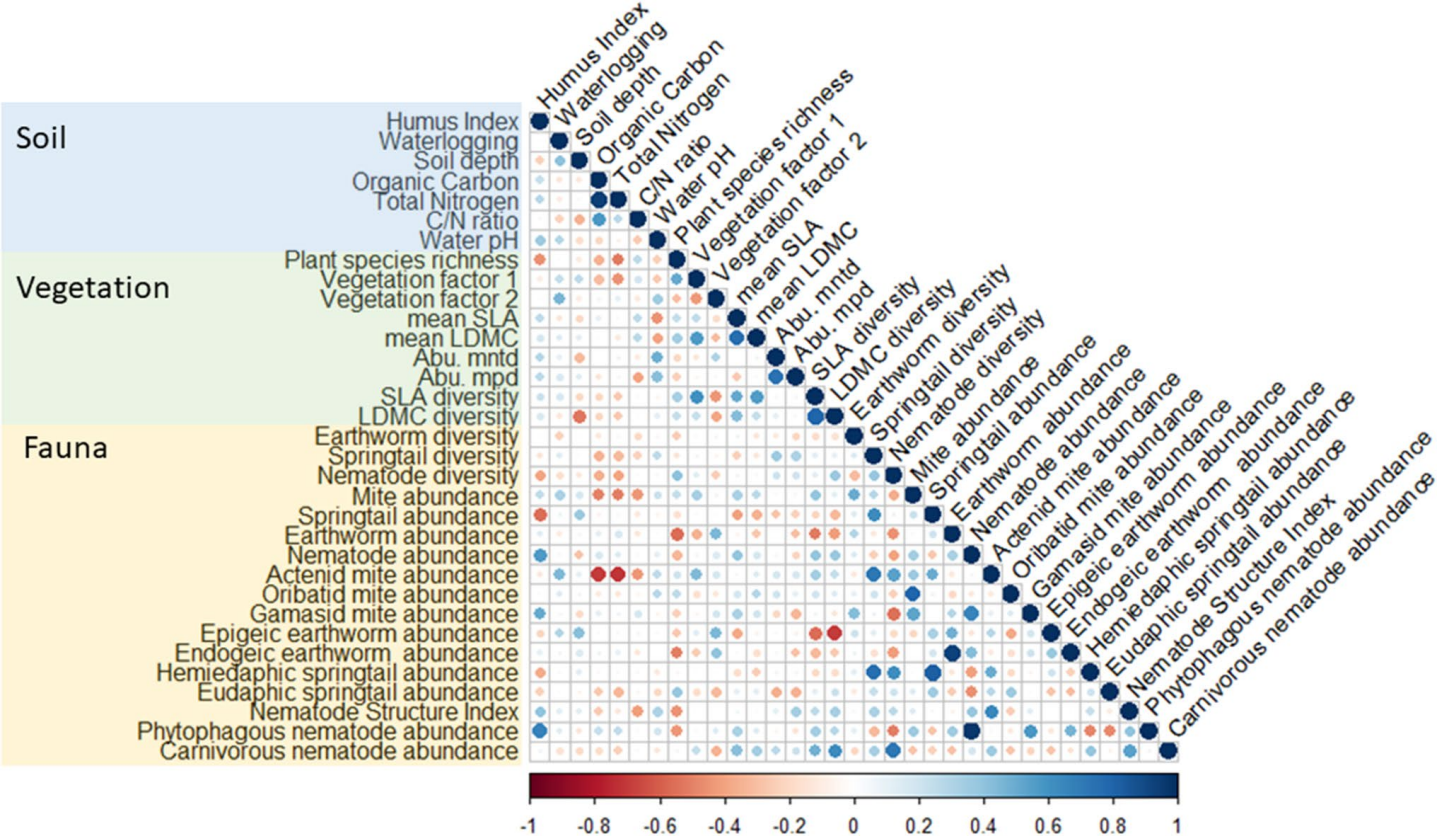
Pearson’s correlations between soil parameters, vegetation parameters, and soil fauna parameters. Vegetation factors were obtained using PCA analysis. Abu.mntd = abundance-weighted mean nearest taxon distance. Abu.mpd = Abundance-weighted mean phylogenetic distance, referred to as phylogenetic diversity in the results, abbreviated as PhylDiv in Table 1. Smaller *p* values are depicted with larger circles. Abundances were log-transformed before analysis. Details can be found in Supplement S3

Plant community composition was summarized using Principal Component Analysis, extracting the two main factors for each site. To calculate phylogenetic diversity, we obtained phylogenetic distances (in millions of years) among the species from the higher plant phylogeny database Daphne (Durka and Michalski 2012). Some plants were identified only to genus level, but were then replaced by a species taken randomly from the same genus, because there was always only one member of that genus present at a given site. We calculated phylogenetic diversity using the Picante package in R (Kembel et al. 2010; R Core Team 2021) as the average across phylogenetic distances within all pairs of species (Webb 2000). We accounted for the abundance of species using “MPD-abundance” which quantifies abundance-weighted mean phylogenetic distances between pairs of individuals (Abu.mpd; Kembel et al. 2010). To control for variation in species richness, we compared these mean distances to those from a null model produced by reshuffling species across communities. We calculated standardized effect size (SES) values as (observed minus mean-null)/(SD-null), and used SES values in further analyses. We calculated in the same way for each community the abundance-weighted phylogenetic distances within pairs of most closely related species, their averages and the SES of that average. The resulting “mean nearest taxon distances” (Webb 2000) were closely correlated to the above phylogenetic diversity calculated across pairs of species (Abu.mntd; Fig. 2) and we hence limited further analyses to phylogenetic diversity.

To calculate the functional trait diversity of the plant communities, we obtained SLA and LDMC for each species from the LEDA database (Kleyer et al. 2008). When several values were present for one species, the median value was calculated. Not fully identified species were treated as missing values. We calculated single-trait functional diversity for SLA or LDMC using Rao’s quadratic entropy index. The Rao Index expresses the probability that two randomly picked individuals in the community are functionally different. Rao = ∑ p_i_. p_j_. d_ij_ where *p* is the abundance of species i and j in the plot, respectively; d_ij_ the dissimilarity in trait value between the two species (Botta‐Dukát 2005). Trait values were first scaled between zero and one, and then the dissimilarity matrix was calculated as the Euclidean distance between trait values for a pair of species. Thus, the dissimilarity d can range from zero (same trait value) to one (complete dissimilarity between species). Overall, both phylogenetic and functional diversity measures account for the abundance of species. Phylogenetic diversity measures distance in evolutionary time, functional diversity measures distance in a functional space. Otherwise, both measures are technically equivalent (Swenson 2011).

### Sampling and determination of soil fauna

Soil fauna was sampled for each site across multiple subsites to integrate the major small-scale variation of soil fauna. Specifically, soil fauna was sampled 5 m northward from the RMQS sampling plots, in 3 × 34 m plots that were homogeneous in plant cover and soil features (see Cluzeau et al. 2010 for further details). This zone was subdivided into 1 × 3 m sub-plots (Cluzeau et al. 2012). Earthworms were sampled in three sub-plots using protocols developed by Bouché (1972) and adapted by Cluzeau et al. (1999, 2003), where the soil is watered with diluted formalin which drives earthworms up to the surface. Earthworms that emerged at the surface were collected and preserved in 4% formalin. To assess how many earthworms remained in the soil after completion of earthworm extraction, a 0.25 × 0.25 × 0.25 m block of soil was dug out at the centre of each quadrat and spread on a plastic sheet and the remaining earthworms were collected. Species identification was performed using a key based on Bouché (1972). Earthworm species were grouped into three categories; epigeic (living at the surface in litter, no burrows), anecic (making deep vertical burrows and feeding at the surface in litter at night), and endogeic (living fully underground in shallow horizontal, branched burrows) based on Bouché (1972) and OPVT (2013). Diversity and abundance of earthworm species were calculated on the pooled sample. Here and below, species diversity was calculated using the Simpson index (1-D, with values ranging from 0 for no diversity to 1 for high diversity), as it is particularly robust against differences in numbers of animals sampled among sites (Rosenzweig 1995). Note also that Simpson diversity corresponds to a Rao diversity where all species differences are equal (Botta‐Dukát 2005), contributing to the consistency of our measures.

Sampling of springtails (Collembola) and mites (Acari) was done in triplicate using a corer that took a cylinder of soil of 6 cm diameter and 5 cm deep (Block 1966; ISO 2006). Microarthropods were extracted from the soil samples using the high gradient method (Macfadyen 1961), where invertebrates avoid a heat source and move down to fall through a gauze into a cooled collection container. Springtails were identified to species, while mites were identified to suborder. Springtails were identified using Gisin (1960) and later updates (Fjellberg 1998; Hopkin 2007; Potapow 2001; Thibaud 2004). Springtails were classified as euedaphic (living inside the soil), hemiedaphic (living both at the surface and in the soil), and epigeic (living at the surface, being exceptional in our samples) according to Gisin (1943).

Nematodes were sampled from the surface soil layer (0–15 cm) in 32 samples (to capture microspatial heterogeneity; e.g. Delaville et al. 1996) that were then pooled. Nematodes were extracted from about 300 g of wet soil by elutriation with water, followed by an active passage through a cotton wool filter for 48 h. Nematodes were then counted under a binocular microscope. After that, nematodes were fixed with a formaldehyde–glycerol mixture and transferred to mass slides. On average, ca. 200 nematodes were identified per mass slide to family or genus level (Andrássy 1984; Bongers 1994; Siddiqi 2000). We ranked trophic groups of nematodes, according to family membership as in Parmelee and Alston (1986). We also calculated the Structure Index which best reflects the absence of disturbance (Ferris et al. 2001). The Structure Index increases with the presence of long-lived species and of carnivores/omnivores vs. fungivores/bacterivores, and hence is an indicator of stable development of the community.

### Data analysis

We first tested for correlations between soil parameters, plant community parameters, and soil fauna parameters using Pearson’s correlations, using the rcorr function in the R package Hmisc (Harrell 2020), and visualized them using the corrplot package (R Core Team 2021; Wei and Simko 2021). We then tested for a relationship between the diversity in SLA and LDMC, and the phylogenetic diversity of a plant community across our study sites using Ordinary Least Squares regression (OLS) in R (R Core Team 2021). Finally, we tested the relationships between functional-trait and phylogenetic diversities of the local plant communities, and the abundance and species diversity of soil fauna using OLS regression models. Separate models were run with SLA and with LDMC. Because plant community parameters tended to differ within sites between RMQS and RMQS-BioDiv data when both sources were available, the source of the plant community data was included as a factor in the models (where both sources were available we used RMQS plant community data). The average value of SLA or LDMC weighted by abundance was included in the models as predictors alongside plant functional trait diversity, because diversities may change with average values, and average values will capture some of the variation in soil parameters and management across sites (Fig. 2, Supplement 3). To test the predictions in Fig. 1, the interaction between phylogenetic diversity and diversity of the functional trait in question (SLA or LDMC) was included in the models. We tested for associations with overall abundances of earthworms, springtails, mites, and nematodes, and within these groups the abundances of sub-groups considered to be particularly strongly versus particularly weakly exposed to plant diversity. For earthworms and springtails, this was in space (epigeic vs endogeic earthworms, hemiedaphic vs euedaphic springtails), for mites and nematodes the exposure to plant traits was based on diet (mainly herbivorous actinid vs. entirely carnivorous gamasid mites, phytoparasite vs. carnivorous nematodes). For nematodes, we also fitted models with Structure Index as the dependent variable, an indicator of stable development of the community based on life history and trophic position. Abundance data were log-transformed, which generally led to more normally distributed residuals. In addition, we also studied the species diversities of earthworms, springtails, and nematodes. All models were run with scaled predictors using the function ‘summ’ of the R package jtools (Long 2022). For some sites, particular soil fauna parameters were not available. Furthermore, up to three outlier data-points were excluded (based on Q–Q plots). This exclusion permitted to have models that represent almost all but not all data points, rather than models that are biased by one or few data points and do not represent the majority of data points (Quinn and Keough 2002). We note that there were no general problems of residual distribution, only individual outliers that would have been outliers for any possible assumed distribution of residuals. Given small numbers of sites, the results of any single regression analysis must be interpreted with caution, and we hence interpreted relationships only when consistent between plant traits within animal taxon, or among animal taxa for a given plant trait, or both. We checked whether there are cases indicative of too many explanatory variables: no explanatory variable being significant but adjusted *R*^2^ being high. There were none. Moreover, variance inflation factors were below two for all predictors main effects and below three for all interactions). Results were illustrated by plotting simple regression relationships between functional trait diversity of plant communities and soil fauna separately for sites with below- and above-median phylogenetic diversity of the plant community, using the R package ggplot2 (R Core Team 2021; Wickham 2016).

## Results

We identified 91 plant species across 19 permanent meadows (Table S.1). Correlation analysis did not suggest major effects of soil parameters on plant community parameters and only limited effects on soil fauna parameters, notably not on several of those that show strong signals in our below regression analyses: diversities of earthworms, springtails or nematodes, abundances of hemiedaphic springtails and carnivorous nematodes, or nematode structure index (Fig. 2). Vegetation factors 1 and 2 (from principal component analysis of plant community composition) were correlated with diversity of SLA, which thereby represented general patterns in the plant community. Vegetation factor 1 was correlated also with means of LDMC, so that inclusion of mean LDMC in the below regression analyses also accounted for vegetation composition. With the exception of soil pH being correlated with one measure of vegetation phylogenetic diversity (Abu.mntd), neither soil nor vegetation factors were strongly related to measures of phylogenetic diversity (Abu.mntd and Abu.mpd), further reducing the risk of our below regression analyses identifying pseudo-relationships between phylogenetic community composition and soil fauna being in reality attributable to the abiotic or biotic environment*.*

**Fig. 3:**
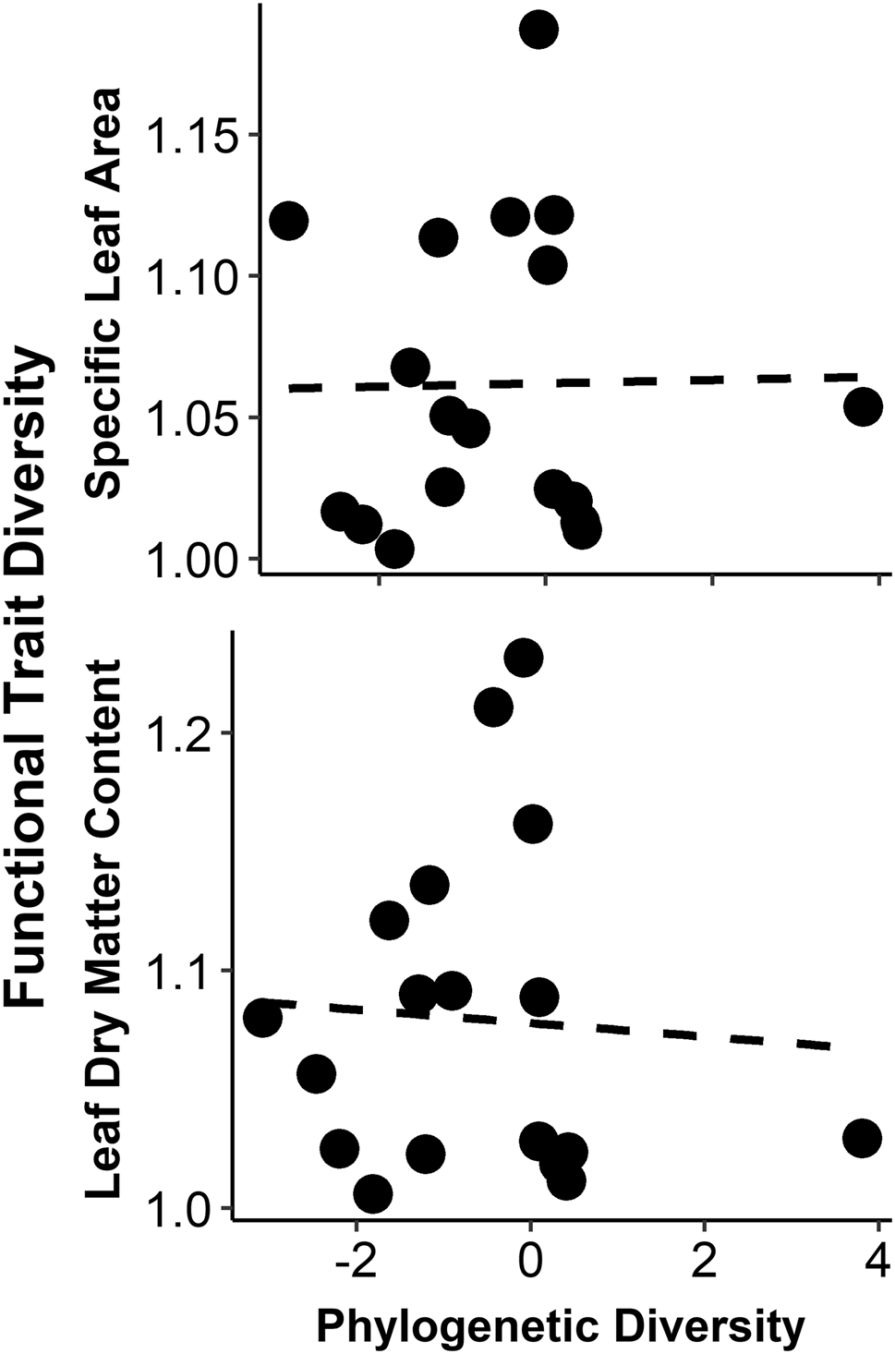
Relationships between diversity of plant functional traits (Y-axis) and plant phylogenetic diversity (X-axis) in permanent meadows in Brittany, France. Regression specific leaf area: *t* = 0.610, *p* = 0.551, *N* = 18, *R*^2^ = 0.022, Regression leaf dry matter content: *t* = 0.596, *p* = 0.560, *N* = 18, *R*^2^ = 0.022, excluding one outlier with extreme phylogenetic diversity

Phylogenetic diversity of plant communities was not significantly related to diversity in plant functional traits (Fig. 3). While phylogenetically uniform plant communities tended to be also uniform in SLA and LDMC, phylogenetically diverse plant communities had trait diversity values for SLA and LDMC that ranged from low to high.

**Fig. 4:**
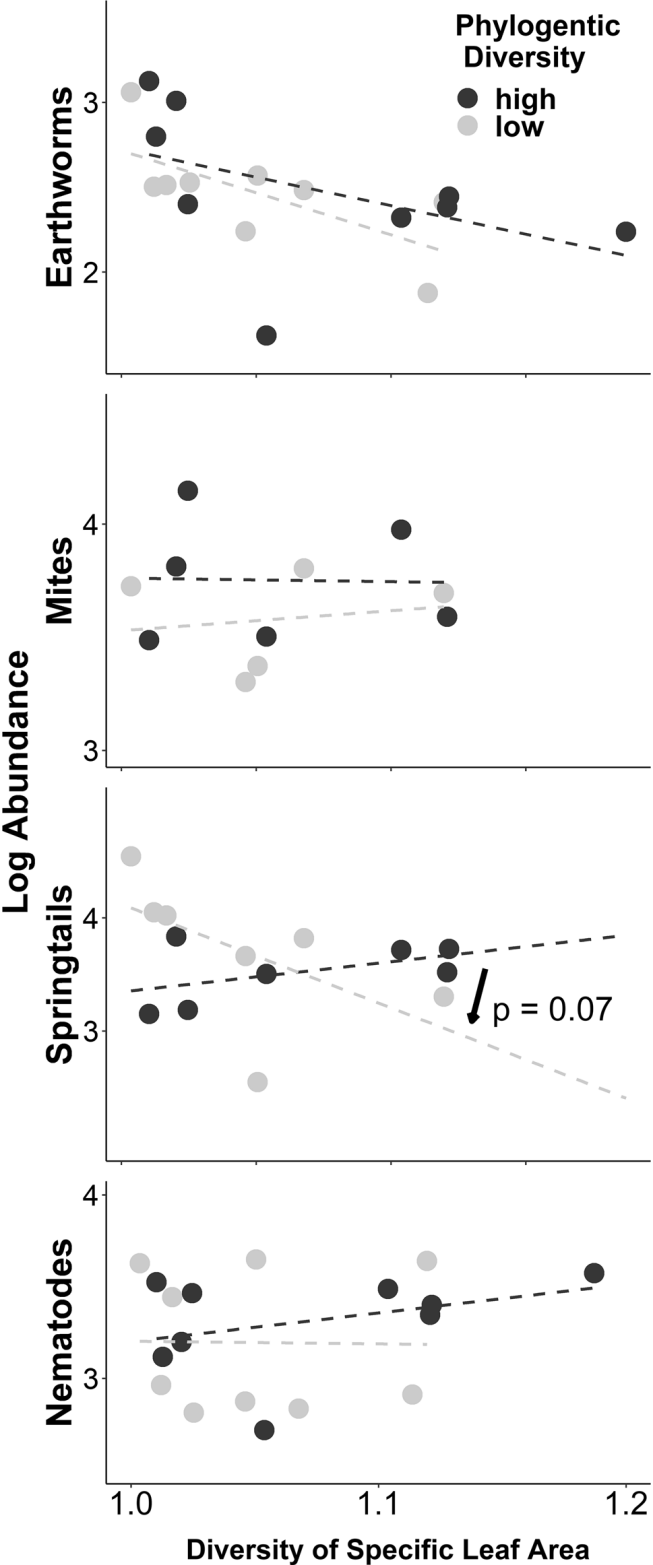
Relationships between the abundance of major groups of soil fauna (Y-axis) and the diversity of specific leaf area (X-axis) for plant communities with below and above-median phylogenetic diversity (< / > − 0.8), illustrating interactions terms listed in Table 1b. The interaction term between diversities is significant in springtails, as indicated by the arrow and p-value in the figure

The abundance of springtails appeared to decline with SLA and LDMC diversity of plant communities when plant phylogenetic diversity is low (Fig. 4, interaction terms in Table 1a). Thus, springtail abundance was higher when plant functional diversity was lower only when plant phylogenetic diversity was low.

**Fig. 5:**
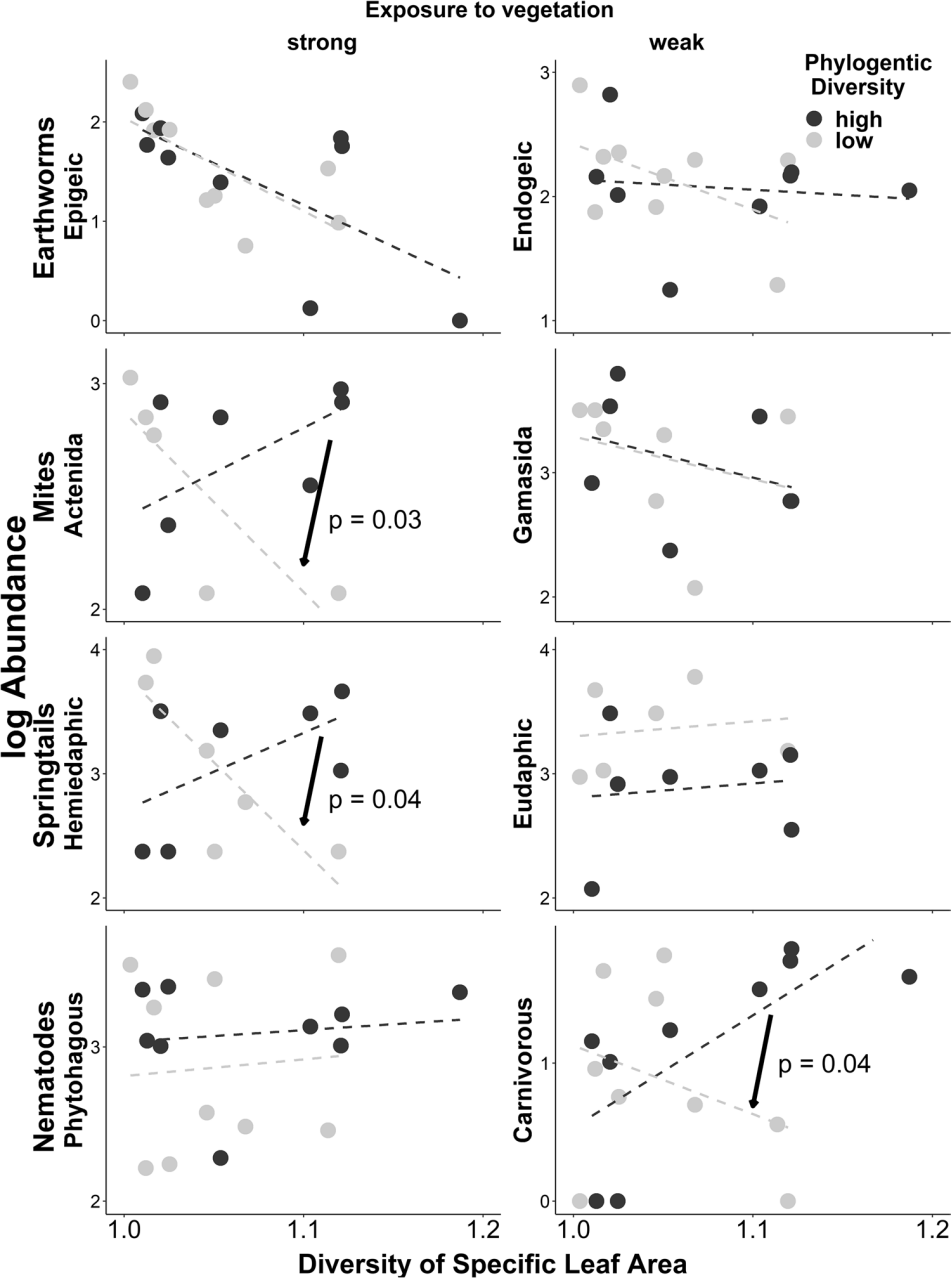
Relationships between the abundance of sub-groups of soil fauna (Y-axis) and the diversity of specific leaf area (X-axis) for plant communities with below and above-median phylogenetic diversity (< / > − 0.8). Sub-groups are strongly (left column) exposed or weakly (right) exposed to vegetation, and hence likely or unlikely to respond to plant community diversity. Exposure is due to spatial position (epigeic vs endogeic earthworms, hemiedaphic vs eudaphic springtails), or diet (plant-feeding acteneid vs carnivorous gamasid mites, phytophagous vs carnivorous nematodes). Figures illustrate interaction terms as listed in Table 1c, d, and e, respectively, significant interaction terms are indicated by arrows and p-values in the figure

Sub-groups of springtails that live spatially exposed to plants in upper soil strata responded to plant community diversity, contrary to groups dwelling in deeper strata. Specifically, abundance of hemiedaphics decreased with plant functional diversity (of SLA and LDMC) when plant phylogenetic diversity was low (see interaction terms in Table 1c, Fig. 5), while abundances of eudaphics did not show such change. A similar trend was observed for mites that are often plant-feeding (actinedids) and SLA, while for LDMC, the interaction of plant phylogenetic and functional diversity was only marginally significant, and similar for both groups of mites (actinedids and gamasids, Table 1d, Fig. 5). For earthworms, no such effects were observed, and for nematodes the pattern was opposite to expected: the interaction was significant for carnivores but not phytophages (Table 1e, Fig. 5). Among nematodes, we also tested whether groups indicative of undisturbed soils (long-lived, higher trophic level, as summarized by Structure Index) mostly responded to plant community diversity, contrary to groups that are indicative of disturbed soils. The Structure Index showed a significant interaction between plant phylogenetic and functional diversity for SLA (Table 1f). When plant phylogenetic diversity was low, lower plant functional diversity was associated with lower values for the nematode Structure Index. The pattern found in Structure Index was distinctly stronger than that for carnivorous nematodes (*F* = 15.9 vs. 11.5, Table 1e, f), suggesting that additionally accounting for life-history information in the Structure Index is pertinent.

**Fig. 6:**
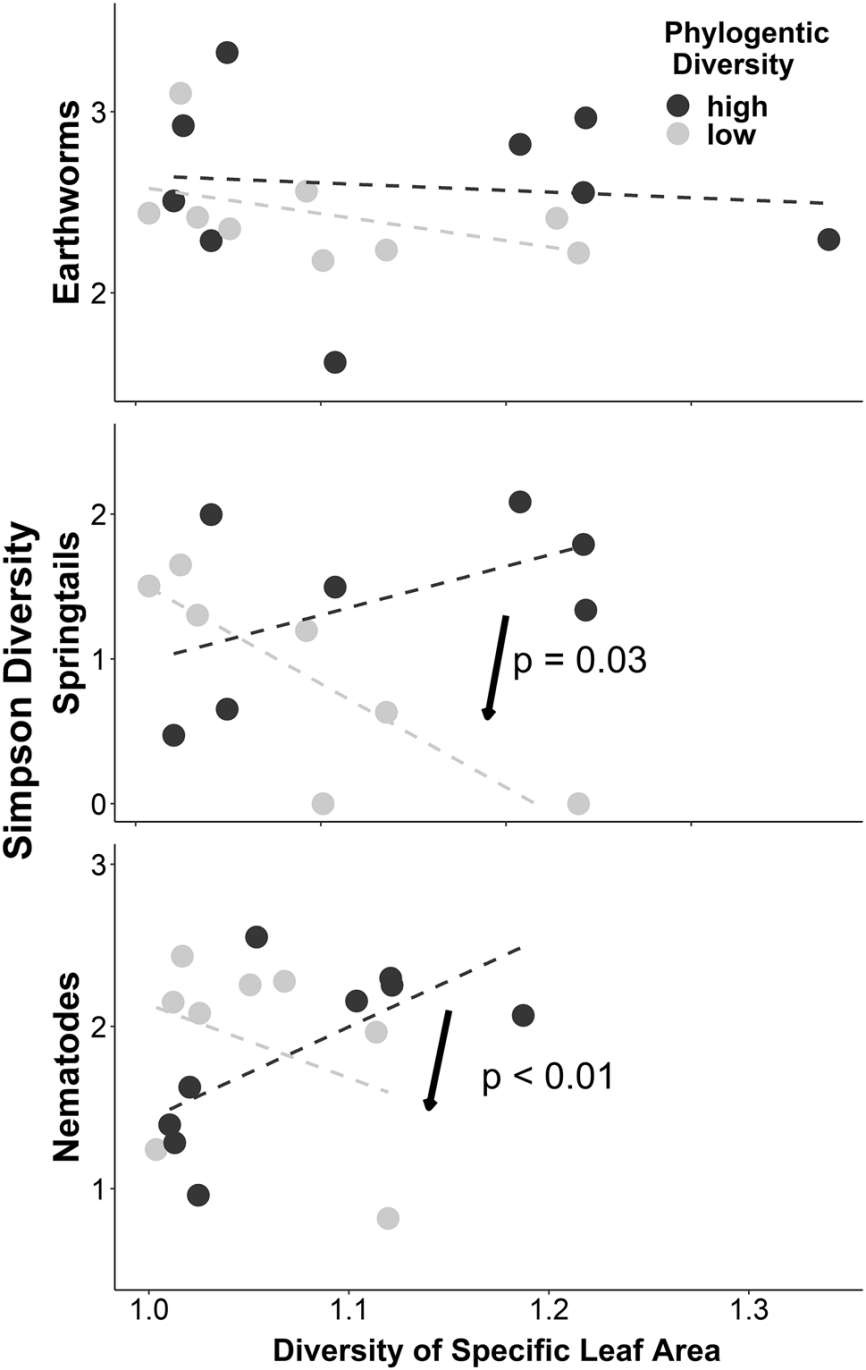
Relationships between the diversity of soil fauna (Y-axis) and the diversity of specific leaf area (X-axis) for plant communities with below and above-median phylogenetic diversity, illustrating interaction terms listed in Table 1g. Significant interaction terms are indicated by arrows and p-values in the figure

Taxonomic diversity of soil fauna declined with trait diversity of plant communities when plant phylogenetic diversity is low. Specifically, higher diversity in SLA was associated with lower diversity of springtails and nematodes when plant phylogenetic diversity is low, while higher diversity in SLA was associated with increased or unchanged diversity of springtails and nematodes when plant phylogenetic diversity was high (interaction terms in Table 1g, Fig. 6). For earthworms, no significant statistical effects of SLA diversity were detected (interaction terms in Table 1g, Fig. 6). Higher diversity in LDMC was associated with lower diversity of earthworms and springtails when plant phylogenetic diversity was low, while higher diversity in LDMC increased diversity of earthworms and springtails when plant phylogenetic diversity was high (interaction terms in Table 1g). For nematodes, no effect of LDMC diversity was detected (interaction terms in Table 1g).

## Discussion

We combined plant community data with soil fauna data for permanent meadows in Brittany, France, and found interactions between phylogenetic and functional-trait diversity of plant communities in determining multiple aspects of the soil fauna. To our knowledge, this is the first time that such interactive effects have been tested, permitting us to explore novel hypotheses on the consequences of phylogenetic lability of a functional trait for how the local diversity of this plant trait within a community drives associated biota. Overall, we found that plant phylogenetic diversity and the diversity of functional traits combined have major power of explaining abundances of several soil fauna groups and of diversity of soil fauna. In most cases, significant effects were detected for those groups that we considered to be particularly exposed to the plants: living close to the plants, or feeding directly on the plants. Importantly, one form of diversity not just complements the other—the interaction terms between phylogenetic and functional trait diversity are also important. These statistical interactions reflect the local assembly of different phylogenetic histories of traits. We found that communities representing trait divergence among close relatives often have reduced abundances and diversities of soil fauna, compared to communities representing close relatives that have similar functional trait values. Our study might hence contribute to identifying the possible local consequences of trait shifts among phylogenetically closely related species.

Our study has several limitations. First, it is a correlational study. We cannot prove causality. In particular, diversities might reflect environmental conditions not accounted for and possibly being the true underlying causes of the patterns observed. We tested for such relationships of diversities to environmental conditions and found only few (Fig. 2). Plant species composition correlated with the diversity of SLA, but not with the diversity of LDMC. Nevertheless, diversities of both functional traits showed similar interaction terms with plant phylogenetic diversity in determining soil fauna. Also, our study does integrate multiple environmental conditions by accounting for community-weighted means of SLA and LDMC as these trait means tend to vary with environmental conditions (Bisigato et al. 2015; Daou et al. 2021; Kichenin et al. 2013; Reich et al. 1999). In our data, these trait means were of much lower statistical importance than diversities and their interactions. Second, our study focused on two important functional traits, but other functional traits such as carbon:nitrogen ratio may also be important. However, these other traits may be related to the traits we considered (Wright et al. 2004). Third, we did not have sufficient data to test for non-linear relationships, but data visualization did not indicate that these are important here. Fourth, our study does not permit to identify effects of diversity that operate through individual plasticity or within-population variation, but focuses on the effects of sorting of species with certain traits into communities. Future studies could sample sites multiple times for vegetation and soil fauna and measure plant traits directly, rather than rely on a database (Fujii et al. 2020; Ganault et al. 2021). Fifth, our results on plant phylogenetic diversity might be contingent on the particular phylogenetic lineages present in this system. Meadows are dominated by grasses, and grasses tend to favor closely related neighbors (Cahill et al. 2008). In addition, combinations of litters of different grass species tend to decompose faster than combinations of grasses with non-grasses (Barbe et al. 2018), consistent with our observation of increased diversity and often abundance of soil fauna with a combined decrease in phylogenetic and trait diversity. Therefore, while for the lineages present in grassland, our results are consistent with the literature, future work on different, non-grass-dominated systems is needed to identify the generality of our results. Sixth, soil fauna will also be affected by biomass removal such as by mowing and grazing (Galvánek and Lepš 2012; Liu et al. 2017; Todd et al. 1992), for which we have insufficient data for our study sites. Even if hay is exported, the local vegetation composition drives the local litter composition. In grasslands, between 50 and 90% of plant primary production ends up as litter (Cebrian 1999).

### Explaining interaction terms between plant phylogenetic and functional diversity

Overall, for the abundances of the major groups of soil fauna, we found a significant and positive effect of the interaction term between plant phylogenetic and functional diversity in the respective subgroups that are particularly exposed to the plants due to their vertical distribution (within earthworms and springtails), diet (within mites but not nematodes), or life span (within nematodes). Only in earthworms did we find that exposure to plants (in epigeic forms) did not increase the interaction between phylogenetic and trait distance. It may be that earthworms can avoid exposure to vegetation by constructing niches as they construct tunnels and may forage at the surface during the night*.* For the diversity of all three major groups of soil fauna tested, we found a significant and positive effect of the interaction between plant phylogenetic diversity and the diversity of at least one functional plant trait. These positive interaction effects are consistent with both hypotheses 1a and d in Fig. 1: plant phylogenetic diversity reinforcing either complementarity of resource traits or dilution of preferred resource traits. We will discuss below which of these hypotheses has more support.

Plant phylogenetic diversity reinforcing complementarity of resource traits (Fig. 1a): a positive interaction term phylogenetic*functional-trait diversity might reflect an increase of resource complementarity due to increasing diversity of the functional trait with increasing plant phylogenetic diversity (Eisenhauer 2012). High diversity of a given functional trait may provide complementary resources for soil fauna only if represented by phylogenetically distant species, and not by phylogenetically closely related species diverging only in a single or few traits. Soil organisms may benefit from such complementarity of multiple resources (consistent with Barbe et al. 2018). For instance, phylogenetically more diverse plant communities might select for a higher proportion of generalist soil fauna that benefit more from resource complementarity due to a more diverse functional trait (for herbivores: Castagneyrol et al. 2014; Grandez-Rios et al. 2015). Moreover, it might be impossible for soil fauna to profit from the diverse values of a given trait if the differences in that trait are not integrated with differences in other plant traits (Pigliucci 2003). Such phenotypic integration of traits seems to be the rule in plants due to trade-offs or allometries, and different phylogenetic lineages of plants occupy different positions along these axes of phenotypic integration (Pigliucci 2003). Soil fauna might have evolved solutions to these phylogenetically conserved, integrated combinations of traits, but not to disintegrated combinations of traits that recently diverged while other traits remained phylogenetically conserved (Alonso and Herrera 2003 but see; Damián et al. 2020 on integrated defenses). A diversity of values of one trait would hence not permit the establishment of a diversity of resource specialists. This scenario of Fig. 1a, however, is unlikely to be the major explanation of the patterns we found. First, we hardly observed high trait diversity for low plant phylogenetic diversity (contrary to Prinzing et al. 2008). Low plant phylogenetic diversity hence usually cannot cancel out the effect of high diversity of a given trait. Second, the main effects of phylogenetic and trait diversity were negative, contrary to predictions of resource complementarity as presented in Fig. 1a.

Plant phylogenetic diversity reinforcing dilution of preferred resource traits (Fig. 1d): A positive interaction term phylogenetic*functional trait diversities on soil fauna might also reflect an increase of resource dilution due to trait diversity with increasing plant phylogenetic diversity, or in other words, an increase of resource concentration due to trait uniformity with increasing phylogenetic uniformity (Root 1973). Low diversity of a given trait may increase the resource concentration for soil fauna only if represented by phylogenetically proximate species, and not by phylogenetically distant species converging in only a single or few traits. Again, soil fauna might have evolved solutions to integrated combinations of traits that have been phylogenetically conserved, but not to disintegrated combinations of traits that recently converged while others remained phylogenetically conserved (Alonso and Herrera 2003; Damián et al. 2020). Low trait diversity of only one or few traits would not increase resource concentration for soil fauna specialized on an integrated multi-trait plant phenotype characteristic for a particular plant lineage. Phylogenetically diverse litters that are uniform in SLA or LDMC might possibly be a mosaic of phylogenetically conserved and recently converged traits, and only few soil-fauna species might be capable of using such a trait mosaic (Pan et al. 2015b) if there has been little evolutionary time to adapt to it. This scenario of soil fauna profiting from resource concentration (Root 1973) only under both trait and phylogenetic uniformity is likely to be the major explanation for the patterns we found. First, we did observe that low trait diversity could occur for both low and high plant phylogenetic diversity. High plant phylogenetic diversity could hence potentially cancel out the effect of low diversity of a given trait. Second, the main effects of phylogenetic and trait diversity were negative, consistent with predictions of resource concentration as presented in Fig. 1d.

### Why uniformity of resources sometimes promotes consumer diversity and sometimes not

The scenario of plant community uniformity increasing soil fauna through resource concentration is consistent with parts of the literature (Barbe et al. 2018; Pan et al. 2015a) but not with others (Milcu et al. 2013). Even within our own study, some results were inconsistent: uniformity of a trait across phylogenetically uniform plant species did not always correspond to increased soil fauna diversity. Inconsistency may result from the idiosyncratic responses of different taxa of soil fauna to different traits of the plant community, and from reinforcement of these idiosyncrasies by particular traits of soil fauna such as vertical distribution, diet, or life span. Other factors like study system may come on top. Such effects would explain why low diversity of a plant community does not always promote diversity of soil fauna or performance of soil fauna (Ganault et al. 2021; Hooper et al. 2000; Wolters et al. 2000).

### Why accounting for phylogenetic diversity advances our understanding of ecosystems

Phylogenetic diversity has been related to ecosystem functioning by multiple authors (Cadotte et al. 2008; Flynn et al. 2011; Narwani et al. 2013; Venail et al. 2015; Yguel et al. 2016), often arguing that phylogenetic diversity might serve as a proxy for the diversity of functional traits (see for critical discussion Cadotte et al. 2008; Gerhold et al. 2015; Srivastava et al. 2012). These authors tend to find no relationships when applying measures of phylogenetic diversity that are independent of species richness (like ours; Narwani et al. 2013; Venail et al. 2015; Yguel et al. 2016). We here use the information on both trait diversity and phylogenetic diversity of plants, to infer scenarios of phylogenetic trait lability or trait conservatism across the species locally assembled into a community. We hence move from using phylogenetic diversity as a proxy for trait diversity to phylogenetic diversity as a tool to interpret the evolutionary origin of trait diversity (as suggested by Prinzing 2016). High functional diversity may sometimes be of evolutionary recent origin due to local assembly of closely related species that have diverged in trait states. Similarly, low functional diversity may be due to the assembly of distantly related species that have converged in trait states. We show that such low trait diversity of recent origin may be particularly disadvantageous for soil fauna and thereby likely also for soil food-webs and decomposition. We further develop this point below.

### Do feedbacks between trait evolution and ecosystem processes exist?

Our results might also have implications for understanding eco-evolutionary feedbacks. Diverse and abundant soil fauna have often been shown to improve litter decomposition (Heemsbergen et al. 2004) and thereby potentially plant growth. The present study suggests that soil fauna diversity and abundance may be low in a plant community in which key functional traits are diverse but phylogenetic lineages are uniform (a community that is composed of close relatives that have recently diverged in the respective traits). Equally, plant communities composed of distant relatives that have converged in traits could be associated with low abundance and diversity of important groups of soil fauna. It can be speculated that low abundance and diversity of soil fauna then reduce litter decomposition rate. Reduced litter decomposition, in turn, might be to the detriment of the plants that produced this litter (Hooper et al. 2000). This (still highly speculative) reasoning suggests feedback between the recent macroevolution of plant traits, the ecological assembly of decomposers, the recycling of nutrients in ecosystems, and the performance of plants: recent phylogenetic lability of a trait has the potential of reducing soil fauna, litter decomposition, and the performance of plants, hence feeding back negatively on itself (Barbe et al. 2020). Such negative feedback might be particularly frequent in disturbed habitat types given that they show a particularly strong pattern of phylogenetic lability of traits (Prinzing et al. 2021). Our study hence contributes to exploring the interface between evolution and ecosystem functioning at an intermediate scale of “recent macroevolution” (evolutionary lability of functional traits among species). This scale is so far still little treated (but see Yguel et al. 2016) compared to now classical approaches relating ecosystem functioning to overall macroevolution as represented by phylogenetic diversity (Cadotte et al. 2009), or to microevolutionary local adaptations within species (Harmon et al. 2019).

## Conclusions

Our results suggest that soil fauna only profits from resource concentration when both the diversity of key plant functional traits and plant phylogenetic diversity are low. This is the case in plant communities characterized by the co-occurrence of closely related plant species that have conserved trait values, and not in plant communities consisting of distantly related plant species that have converged in values of the key functional trait. Trait evolution across plant lineages and the local assembly of these traits and lineages might drive the abundance and diversity of soil fauna, which in turn control the recycling of plant litter and thereby potentially influence the performance of the plants.

## Supplementary Information

Below is the link to the electronic supplementary material.

Supplementary file1 (XLSX 140 KB)

## Data Availability

We propose to make the metadata accessible as a supplementary file with metadata and correlation matrix.
